# Supplementary figures and images for: Apparent source levels and active communication space of whistles of free-ranging Indo-Pacific humpback dolphins (Sousa chinensis) in the Pearl River Estuary and Beibu Gulf, China
Source: PeerJ. 2016 Feb 15;4:e1695. doi: 10.7717/peerj.1695 (PMC4756734; doi:10.7717/peerj.1695)

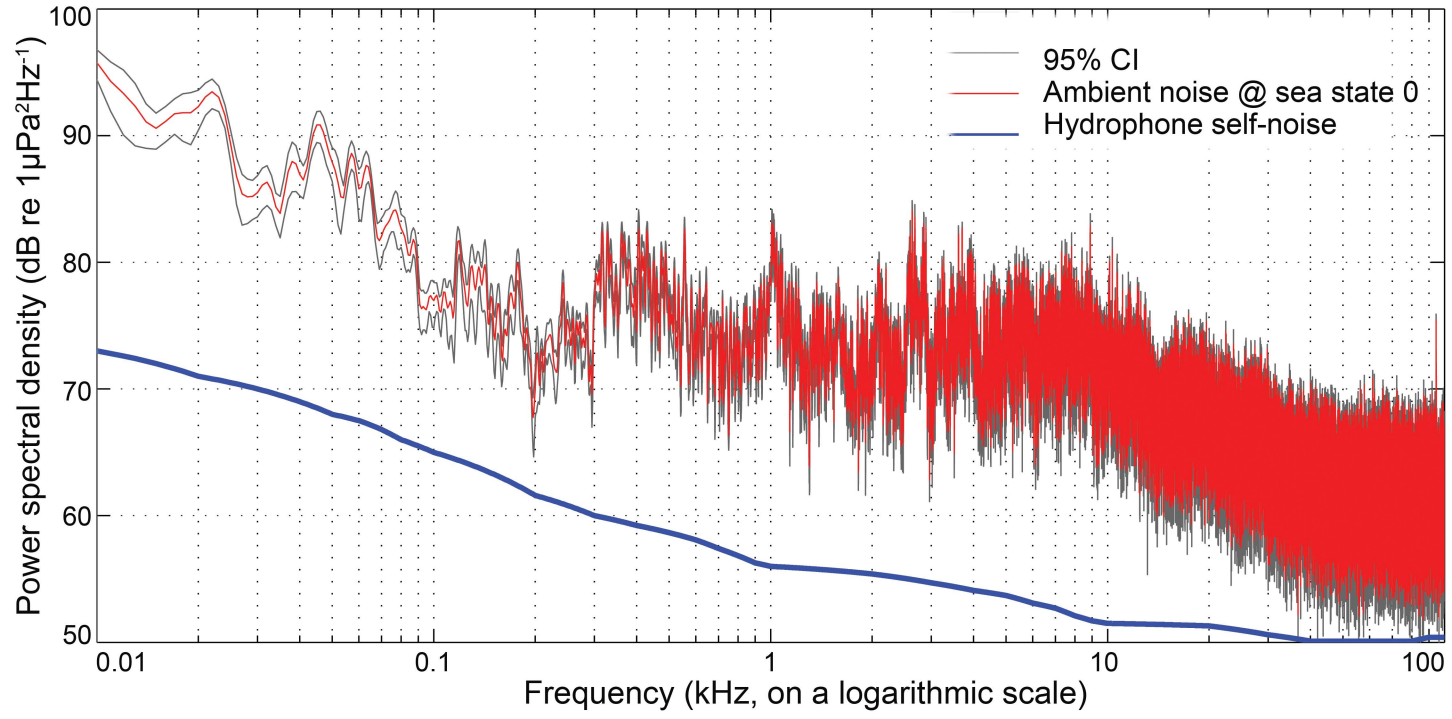

Supplement: Figure S1 — The nominal self-noise level was obtained when the hydrophone was connected to the voltage pre-amplifier VP2000 and was provided courtesy of the Reson company. The gray lines were the 95% confidence interval of the ambient noise. [file peerj-04-1695-s001.pdf]

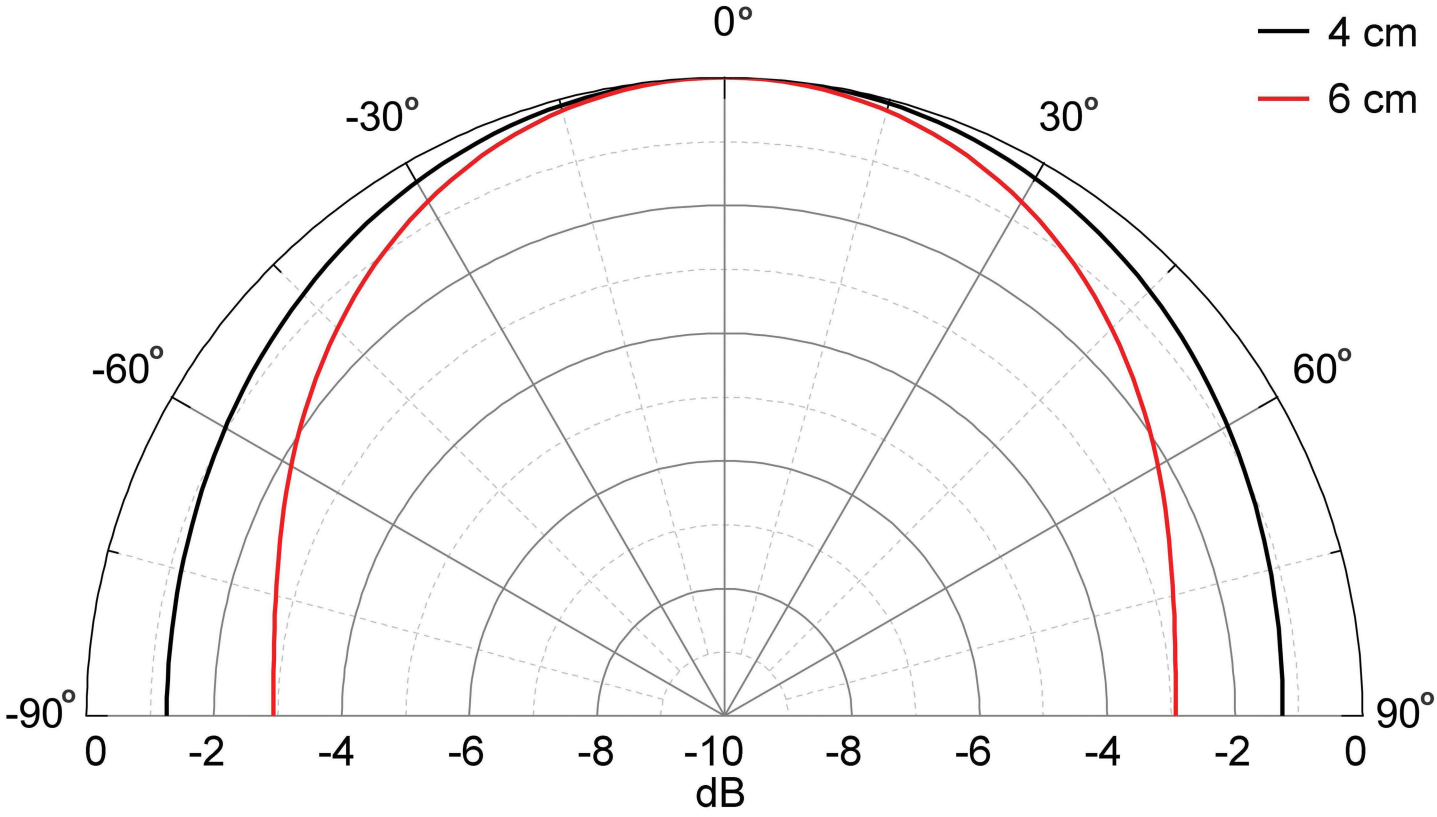

Supplement: Figure S2 — Modeling was based by using a radius of 4 cm (directivity index = 3 dB) and 6 cm (directivity index = 6 dB) piston transducer at typical Sousa whistle frequency of 6.35 kHz. [file peerj-04-1695-s003.pdf]
