# Supplementary material for: Apparent source levels and active communication space of whistles of free-ranging Indo-Pacific humpback dolphins (Sousa chinensis) in the Pearl River Estuary and Beibu Gulf, China
Source: PeerJ. 2016 Feb 15;4:e1695. doi: 10.7717/peerj.1695 (PMC4756734; doi:10.7717/peerj.1695)
Supplement: File S1 [file peerj-04-1695-s002.docx]

The sound propagation model was based on the assumptions that the water surface and the sediment bottom were flat, and the sound-speed profile at each field site was homogeneous. A test signal, assumed to be the source signal, was imported into the model and the received sound with animal, i.e., sound source, at difference distance between hydrophone and animal and different animal depth were estimated for each hydrophone depth. The animal depth ranges from the water surface (0) to the bottom at 1 m increments and the distance between hydrophone and animal was start at 1 m and with an increment of 1 m. The signal at the receiver was estimated as the sum of the direct path signal and multipath propagated signal after accounting for absorption, refraction, and reflections from the surface and or bottom boundaries [[1](#_ENREF_1)] using following equations:

$S_{r}\text{=}S_{dp}\text{+}\sum_{m=1}^{j} S_{sp}(m)+\sum_{m=1}^{j} S_{bp}(m)$ (1)

Where :

$S_{dp}\text{=}S(t)/r_{0}$ (2)

$S_{sp}(m)=S(t-\Delta t_{sp}(m))\times R_{s}(m)^{ns_{sp}(m)}\times R_{b}(m)^{nb_{sp}(m)}/r_{s}(m)$ (3)

$S_{bp}(m)=S(t-\Delta t_{bp}(m))\times R_{s}(m)^{ns_{bp}(m)}\times R_{b}(m)^{nb_{bp}(m)}/r_{b}(m)$ (4)

 (5)

 (6)

$\Delta t_{sp}(m)=(r_{s}(m)-r_{0})/c_{w}$ (7)

$\Delta t_{bp}(m)=(r_{b}(m)-r_{0})/c_{w}$ (8)

$r_{s}(m)=\sqrt{dAa^{2}+h_{s}(m)^{2}}$ (9)

$r_{b}(m)=\sqrt{dAa^{2}+h_{b}(m)^{2}}$ (10)

$h_{s}(m)=m\times dw+da-dh$ (11)

$h_{b}(m)=m\times dw-da+dh$ (12)

$ns_{sp}(m)=nb_{sp}(m)=ns_{bp}(m)=nb_{bp}(m)=m/2$ (13)

if m was even, or:

$h_{s}(m)=(m-1)\times dw+da+dh$ (14)

$h_{b}(m)=(m+1)\times dw-da-dh$ (15)

$ns_{sp}(m)=nb_{bp}(m)=(m+1)/2$ (16)

$nb_{sp}(m)=ns_{bp}(m)=(m-1)/2$ (17)

If m was odd, and where *S_r_* was the signal at the receiver, *S_dp_* was the direct path signal, *S_sp_* was defined as the multipath propagation signal with the initial reflection point at the air–water interface, *S_bp_* was defined as the multipath propagation signal with the first reflection point at the water–bottom interface, *m* was the total number of signal reflection points at the air–water and water–bottom interfaces for a multipath propagation signal, *j* was the maximum reflection boundary in consideration and was conservatively defined as 20 in this study. *S(t)* was the signal at the source in volts, *r_0_* was the length of the direct sound path in m, *Rs(m)* and *Rb(m)* were the reflective [coefficient](javascript:void(0);)s at the air–water interface and water-bottom interface respectively, for multipath propagation with total number of m reflection points, *ns_sp_(m)* and *nb_sp_(m)* were the total number of reflections at the air–water interface and water-bottom interface, respectively, for multipath propagation with a total number of m reflection points and the initial reflection point at the air–water interface, *ns_bp_(m)* and *nb_bp_(m)* were the total number of reflections at the air–water interface and water-bottom interface, respectively for multipath propagation signal with a total number of m reflection points and the initial reflection point at the water–bottom interface, *r_s_(m)* and *r_b_(m)* were the signal propagation length for multipath propagation with a total number of m reflection points and the initial reflection point at the air–water and water–bottom interface, respectively. *△t_sp_(m)* and *△t_bp_(m)* were the time lags of the arrival time at the receiver between direct and multipath arrivals with a total number of m reflection points and the initial reflection point at the air–water and water–bottom interface, respectively. *z_s_* , *z_m_* and *z_b_* were the acoustic impedances of the air, water and substance, respectively. *θ_s_(m)* and *Φ_s_(m)* were the incident (same as reflected) and transmitted angle, respectively for multipath propagation signal with a total number of m reflection points and the initial reflection point at the air–water interface (Fig.3). *θ_b_*(m) and *Φ_b_(m)* were the incident (same as reflected) and transmitted angle, respectively, for multipath propagation signal with a total number of m reflection points and the initial reflection point at the water–bottom interface (Fig.3), *c_s_* *c_w_* and *c_b_* are the sound velocity in air, water and substance, respectively, *ro* was the horizontal separation between the animal and the hydrophone, *h_s_(m)* and *h_b_(m)* were the height (vertical propagation length) of the multipath propagation signal with a total number of m reflection points and the initial reflection point at the air–water and water–bottom interface, respectively by referencing the animal location, *dh*, *da* and *dw* were the depth of the receiving hydrophone, the animal and the water, respectively.

*θ_s_(m)* and*Φ_s_(m)* (in the upper left inset of Fig.3) were linked according to Snell’s law:

$\tan[\theta_{s}(m)]=dAa/h_{s}(m)$ (18)

 (19)

*θ_b_(m)* and *Φ_b_(m)* (in the lower left inset of Fig.3) were linked according to Snell’s law:

$\tan[\theta_{b}(m)]=dAa/h_{b}(m)$ (20)

 (21)

In the water-bottom surface, if the incident angle was larger than the critical angle (*θ_c_*), where

$\theta_{c}=\arcsin(c_{w}/c_{b})$ (22)

then the signal will be total reflected, and no real transmitted angle exist, the *R_b_(m)* was set at 1.

Reference

1. Urick RJ (1983) Principles of underwater sound. New York: McGraw-Hill.
